# Laparoscopic excision of accessory spleen for recurrent autoimmune hemolytic anemia after splenectomy: a case report

**DOI:** 10.1186/s40792-024-01884-z

**Published:** 2024-05-03

**Authors:** Ryosuke Kashiwagi, Masaharu Ishida, Koichi Onodera, Shuichi Aoki, Masahiro Iseki, Takayuki Miura, Hideo Ohtsuka, Masamichi Mizuma, Kei Nakagawa, Takashi Kamei, Michiaki Unno

**Affiliations:** 1 Department of Surgery, Tohoku University Graduate School of Medicine, 1-1 Seiryomachi, Aobaku, Sendai, Miyagi 980-8574 Japan; 2 Division of Hepato-Biliary-Pancreatic Surgery, Shizuoka Cancer Center, 1007 Shimonagakubo, Nagaizumi-cho, Sunto-gun, Shizuoka, 411-8777 Japan; 3 Department of Hematology, Tohoku University Hospital, 1-1 Seiryomachi, Aobaku, Sendai, Miyagi 980-8574 Japan

**Keywords:** Laparoscopic excision of accessory spleen, Autoimmune hemolytic anemia, AIHA, Intraoperative ultrasonography

## Abstract

**Background:**

Splenectomy is indicated in cases of autoimmune hemolytic anemia (AIHA), which are refractory to medical management. In post-splenectomy, there exists a theoretical risk of AIHA recurrence, especially if an accessory spleen undergoes compensatory hypertrophy. In this context, we present a unique case of recurrent AIHA managed through laparoscopic excision of the accessory spleen (LEAS).

**Case presentation:**

A 60-year-old male underwent laparoscopic splenectomy (LS) for AIHA refractory to standard medical therapies. Following the surgery, there was a marked improvement in hemolytic anemia symptoms, and oral steroid therapy was terminated 7 months post-LS. Nonetheless, a year after the LS, the patient exhibited a marked decline in hemoglobin levels, dropping to a concerning 5.8 g/dl, necessitating the reintroduction of oral steroids. A subsequent contrast-enhanced computed tomography (CT) scan unveiled an enlarged accessory spleen. The patient then underwent LEAS, during which the accessory spleen, obscured within adipose tissue, proved challenging to visualize laparoscopically. This obstacle was surmounted utilizing intraoperative ultrasonography (US), enabling successful excision of the accessory spleen. The post-surgical period progressed without complications, and the steroid dosage was reduced to one-twelfth of its initial preoperative quantity.

**Conclusions:**

Recurrent AIHA can be instigated by post-splenectomy compensatory hypertrophy of the accessory spleen. Ensuring comprehensive splenic tissue excision is crucial in AIHA management to obviate recurrent stemming from hypertrophic remnants. In scenarios of AIHA recurrence tied to an enlarged accessory spleen, LEAS stands as a viable and effective therapeutic modality.

## Background

Autoimmune hemolytic anemia (AIHA) is a relatively uncommon condition, manifesting at a rate of 0.8 cases per 100,000 individuals annually. It is characterized by the destruction of red blood cells through autoantibodies directed against antigens on their surface [1, 2]. Although many AIHA patients exhibit symptoms of compensated hemolysis or mild anemia—such as fatigue, dyspnea, and palpitations—coupled with clinical signs, such as pallor and icterus, a subset remains asymptomatic. The primary therapeutic intervention involves corticosteroids, occasionally supplemented with rituximab [1]. For cases refractory to standard medical management, splenectomy has emerged as a potent therapeutic recourse, showing high efficacy [2]. With the evolution of surgical techniques, laparoscopic splenectomy (LS) has been increasingly adopted for hematological disorders [3], becoming the procedure of choice for refractory AIHA. One of the challenges associated with LS is the potential oversight of accessory spleens due to their inconspicuous nature laparoscopically. Such non-excision can, in theory, lead to AIHA recurrence, triggered by compensatory hypertrophy of the remnant accessory spleen [4]. In this context, we present a unique case of recurrent AIHA managed through laparoscopic excision of the accessory spleen (LEAS).

## Case presentation

**Fig. 1:**
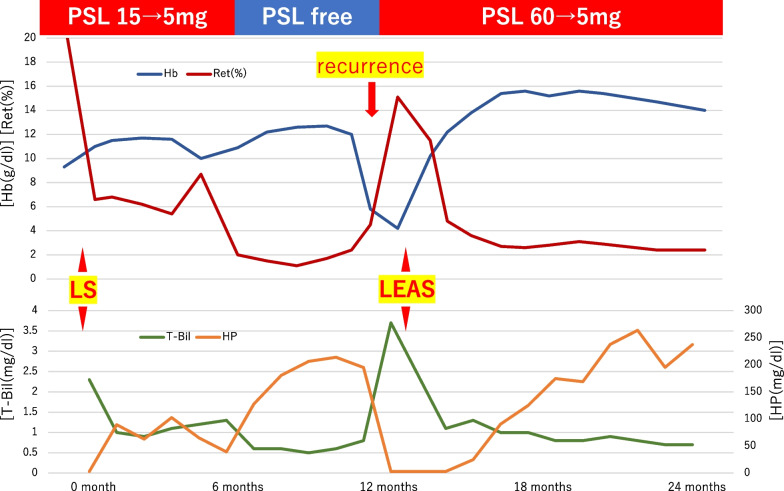
HYPERLINK "sps:id::fig1||locator::gr1||MediaObject::0"Clinical progression timeline: following the initial LS, steroid dosage was tapered from 15 mg/day to 5 mg/day. Anemia re-emerged a year post-surgery, prompting the LEAS procedure. After the second surgery, steroid dosage was tapered from 60 mg/day to 5 mg/day. *Hb* hemoglobin. *Ret* reticulocyte. *T-Bil* total-bilirubin. *HP* haptoglobin. *LS* laparoscopic splenectomy. *LEAS* laparoscopic excision of accessory spleens. *PSL* prednisolone, 60 mg is equivalent to 1 mg/kg for this patient

**Fig. 2:**
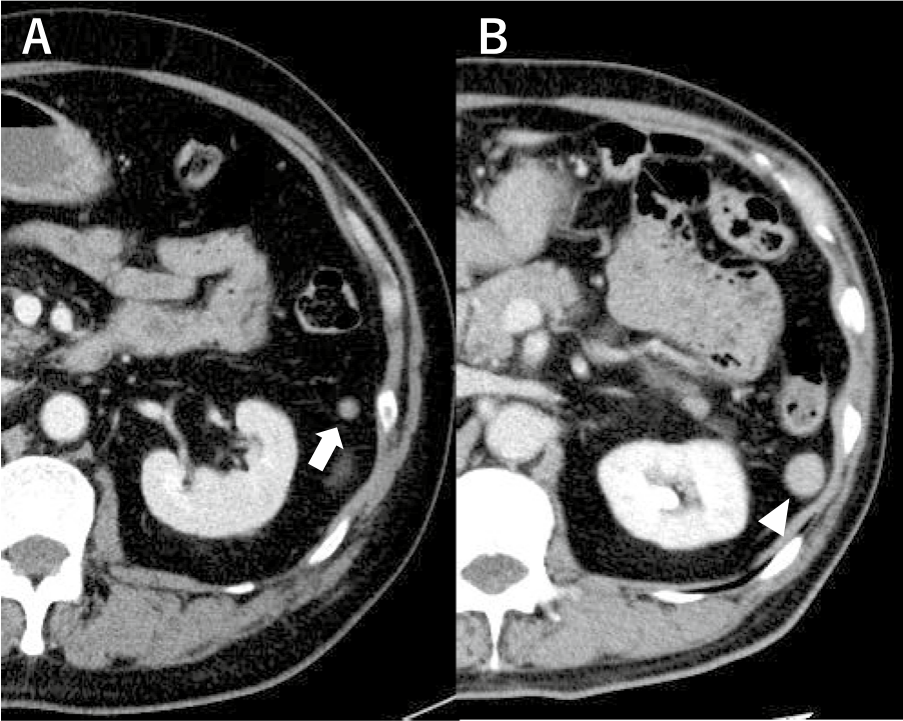
Contrast-enhanced CT scan depicting accessory spleen hypertrophy: prior to the initial LS, the accessory spleen measured 7 mm (arrow). It expanded to 16 mm upon AIHA recurrence (arrowhead). **A** pre-laparoscopic splenectomy; **B** at AIHA recurrence

**Fig. 3:**
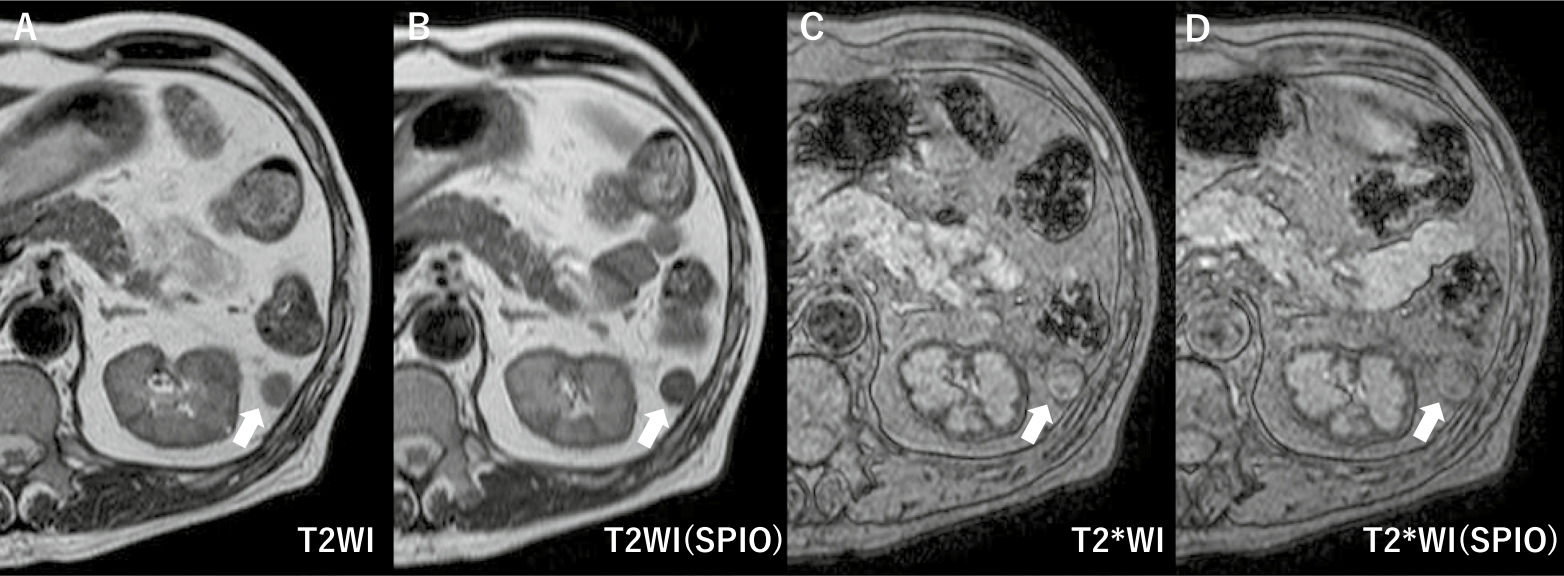
Accessory spleen imaging via SPIO-enhanced MRI: the MRI highlighted a marked signal reduction (arrow) on the SPIO-enhanced T2 and T2* weighted images (WI). **A** T2WI, **B** SPIO-enhanced T2WI, **C** T2*WI, D: SPIO-enhanced T2*WI

**Fig. 4:**
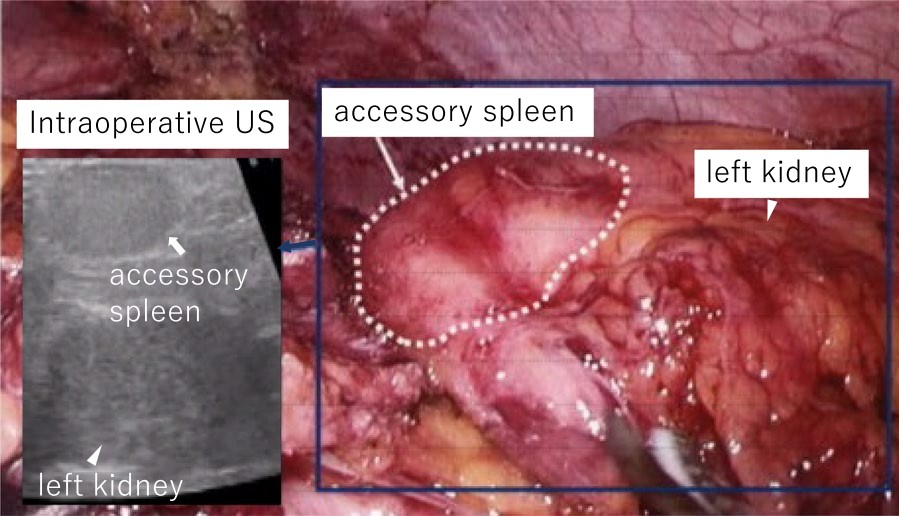
Surgical photograph and intraoperative ultrasonography illustrating the accessory spleen: during the procedure, ultrasonography identified the accessory spleen. Accessory spleen, arrow; left kidney, arrowhead

A 60-year-old male underwent LS for AIHA refractory to standard medical therapies, including oral steroids, steroid pulse, and cyclophosphamide. Following the surgery, there was a significant improvement in hemolytic anemia symptoms, prompting a sequential reduction and ultimate cessation of oral steroids within seven postoperative months (Fig. 1). Nevertheless, a year after LS, the patient's hemoglobin levels precipitously decreased to a critical 5.8 g/dl (hemoglobin 5.8 g/dl, total-bilirubin 3.7 mg/dl, reticulocyte 4.5%, haptoglobin < 3.0 mg/dl). This necessitated the reintroduction of oral steroid therapy (Fig. 1). The contrast-enhanced computed tomography (CT) scan revealed a pronounced hypertrophy of an accessory spleen (Fig. 2). Concurrently, a marked signal attenuation was noted on the superparamagnetic iron oxide (SPIO)-enhanced T2 and T2*-weighted MR images, corroborating the presence of an accessory spleen (Fig. 3). The recurrence of AIHA was considered to be due to the accessory spleen's compensatory hypertrophy, prompting the decision to undertake LEAS. Encased within adipose tissue, the accessory spleen was challenging to discern laparoscopically (Fig. 4). Nonetheless, intraoperative ultrasonography (US) facilitated the successful identification and resection of the accessory spleen (Fig. 4). The surgical procedure required 140 min, followed by an uncomplicated postoperative phase. Histopathological examination verified the resected specimen as splenic tissue. Postoperatively, there was a notable improvement in the hemolytic anemia symptoms. Oral steroid dosage was systematically tapered from 60 mg/day to 5 mg/day, with the patient maintaining remission 3 years since LEAS (Fig. 1).

## Discussion

AIHA is an uncommon autoimmune disorder that produces the antibodies against red blood cell antigens, leading to hemolytic anemia. Although corticosteroid therapy is the primary treatment modality and often yields positive outcomes, many patients eventually become steroid-dependent, making cessation challenging; some patients even require alternative therapeutic approaches. In cases of AIHA refractory to conventional medical management, splenectomy is the recommended course based on its high efficacy rate. After splenectomy, approximately two-thirds of patients exhibit either complete or partial remission in the short term. Furthermore, there is evidence suggesting that a significant portion of these patients will experience sustained remission, negating the need for further medical interventions for prolonged durations [2]. Because of the improved laparoscopic techniques, LS has become the prevalent surgical procedure for patients with hematological disorders necessitating splenectomy [3]. In the presented case, LS was chosen as a secondary intervention for AIHA refractory to standard treatments. The post-LS surgical outcomes were satisfactory until the event of disease recurrence.

The primary objective of splenectomy in hematological conditions is the comprehensive removal of all splenic tissues, including both the main spleen and any accessory spleens. According to the findings presented by Maskal et al., splenectomy resulted in short-term complete remission in 61% of the patient cohort. However, this remission rate diminished to 39% at the 1-year follow-up, with 22% of the patients exhibiting symptomatic recurrence [5]. The incidence of recurrence attributable to the presence of accessory spleens remains indeterminate. Notwithstanding, accessory spleens are detected in 10–30% of the population at autopsy, suggesting a potential impact on the therapeutic outcomes of splenectomy procedures [5, 6]. Targarona et al. underscored the importance of meticulous care during LS for hematological disorders to circumvent parenchymal rupture, cell spillage, and inadvertent retention of accessory spleens, which could culminate in surgical failure [7]. While some studies suggest that preoperative CT scans might be redundant for the detection and localization of accessory spleens, given their ostensible visibility adjacent to the spleen during laparoscopy [8, 9], Gigot et al. contended that accessory spleens might elude complete identification via laparoscopy alone [4]. In the presented case, the accessory spleen evaded detection during the initial LS and remained challenging to discern during the subsequent LEAS, despite a meticulous search. Consequently, we suggest there should be exhaustive identification of accessory spleens using imaging techniques prior to LS. Tools such as Levovist-enhanced US, CT scanning, Tc-99 m denatured red blood cell scintigraphy, and SPIO-enhanced MRI have demonstrated efficacy in detecting accessory spleens [10].

During LEAS, an accessory spleen, ensconced within adipose tissue, presented challenges via laparoscopy in our patient. Supplementary techniques such as employing a handheld gamma probe and preoperative CT-guided methylene blue injection have been recommended to enhance laparoscopic explorations and ensure that no accessory spleens remain undetected [11, 12]. Remarkably, in our instance, intraoperative US proved invaluable in delineating an accessory spleen, despite infrequent mention in existing LEAS literature. Intraoperative US offers a straightforward, safe, and effective approach to locate accessory spleens, proving to be particularly beneficial in scenarios marked by previous surgical adhesions or when the accessory spleens are shrouded by adipose tissue, as observed in our patient.

In our case, the recurrence of AIHA was linked to the presumed compensatory hypertrophy of the accessory spleen that wasn't removed during the initial LS procedure. Certainly, the recurrence of AIHA due to compensatory hypertrophy of the accessory spleen is less prevalent in the medical literature compared to conditions, such as ITP and other hematological disorders [13, 14]. There exists a singular case report addressing LEAS in the context of AIHA [15], but it lacks an in-depth exploration. Our patient's journey—undergoing LEAS following AIHA relapse post-splenectomy and then exhibiting symptom alleviation—adds a significant case to the existing literature.

Given the insights from our case, we describe three key recommendations:

- **Concurrent Resection**: When performing splenectomy for AIHA, there should be a conscious effort to locate and excise accessory spleens concurrently to minimize the risk of recurrence.
- **Thorough Investigation for Recurrence**: In cases where AIHA manifests again following a splenectomy, an intensive evaluation targeting the presence of an accessory spleen is essential. The act of resecting such an accessory spleen may offer alleviation from hemolytic manifestations.
- **Utility of Intraoperative US in LEAS**: Particularly when the accessory spleen is concealed by adipose layers, intraoperative US emerges as a practical tool during the LEAS procedure to ensure accurate detection.

## Conclusions

Recurrent AIHA can be instigated by post-splenectomy compensatory hypertrophy of the accessory spleen. Ensuring comprehensive splenic tissue excision is crucial in AIHA management to obviate recurrence stemming from hypertrophic remnants. In scenarios of AIHA recurrence tied to an enlarged accessory spleen, LEAS stands as a viable and effective therapeutic modality.

## Data Availability

All data generated or analyzed during this study are included in this published article.

## Abbreviations

AIHA: Autoimmune hemolytic anemia
LEAS: Laparoscopic excision of accessory spleen
LS: Laparoscopic splenectomy
US: Ultrasonography
CT: Computed tomography
SPIO: Superparamagnetic iron oxide
